# Paving Therapeutic Avenues for FOXG1 Syndrome: Untangling Genotypes and Phenotypes from a Molecular Perspective

**DOI:** 10.3390/ijms23020954

**Published:** 2022-01-16

**Authors:** Ipek Akol, Fabian Gather, Tanja Vogel

**Affiliations:** 1 Department of Molecular Embryology, Institute for Anatomy and Cell Biology, Medical Faculty, University of Freiburg, 79104 Freiburg, Germany; ipek.akol@anat.uni-freiburg.de (I.A.); fabian.gather@anat.uni-freiburg.de (F.G.); 2 Faculty of Biology, University of Freiburg, 79104 Freiburg, Germany; 3 Center for Basics in NeuroModulation (NeuroModul Basics), Medical Faculty, Albert-Ludwigs-University Freiburg, 79104 Freiburg, Germany

**Keywords:** Rett syndrome, FOXG1 syndrome, neurodevelopmental disorders, disease modelling, hiPSCs, organoids, FOXG1, brain development

## Abstract

Development of the central nervous system (CNS) depends on accurate spatiotemporal control of signaling pathways and transcriptional programs. Forkhead Box G1 (FOXG1) is one of the master regulators that play fundamental roles in forebrain development; from the timing of neurogenesis, to the patterning of the cerebral cortex. Mutations in the *FOXG1* gene cause a rare neurodevelopmental disorder called FOXG1 syndrome, also known as congenital form of Rett syndrome. Patients presenting with FOXG1 syndrome manifest a spectrum of phenotypes, ranging from severe cognitive dysfunction and microcephaly to social withdrawal and communication deficits, with varying severities. To develop and improve therapeutic interventions, there has been considerable progress towards unravelling the multi-faceted functions of FOXG1 in the neurodevelopment and pathogenesis of FOXG1 syndrome. Moreover, recent advances in genome editing and stem cell technologies, as well as the increased yield of information from high throughput omics, have opened promising and important new avenues in FOXG1 research. In this review, we provide a summary of the clinical features and emerging molecular mechanisms underlying FOXG1 syndrome, and explore disease-modelling approaches in animals and human-based systems, to highlight the prospects of research and possible clinical interventions.

## 1. Introduction

FOXG1 syndrome (OMIM #613454) is a rare and severe neurodevelopmental disorder caused by heterozygous de novo mutations in the gene encoding the transcription factor Forkhead Box G1 (FOXG1). FOXG1 has fundamental and non-redundant roles in brain development, from the timing of neurogenesis to the patterning of the cerebral cortex [1,2]. It has been previously classified as a congenital variant of Rett syndrome (RTT, OMIM #312750), due to clinical similarities. Nevertheless, a combination of developmental and anatomical features distinguishes FOXG1 syndrome from the typical Rett syndrome, which is caused by mutations in the X-linked gene encoding for the transcriptional regulator methyl-CpG-binding protein (*MECP2*) [3]. Compared to typical RTT, the FOXG1 syndrome shows an earlier onset in patients, who manifest a complex spectrum of phenotypes, comprising microcephaly, corpus callosum agenesis, delayed myelination, seizures, disrupted circadian rhythm, social withdrawal, and severe intellectual disability, with poor or absent speech development [4,5]. Additionally, FOXG1 syndrome is associated with autism spectrum disorders (ASD) and *FOXG1* variants are identified in patients with ASD [6]. However, due to its variable and broad spectrum of phenotypes FOXG1 syndrome remains underdiagnosed, which consequently limits the research on its etiology and potential therapeutic interventions.

As of October 2021, 860 cases of FOXG1 syndrome have been reported worldwide, with the number of diagnosed individuals increasing as genetic testing becomes more prevalent [7]. Thus far, the identified mutations associated with FOXG1 syndrome have included chromosomal micro-aberrations, such as deletions and duplications, as well as frameshifts and point mutations [4,5,8,9,10]. Depending on the type of mutation, a variability in phenotypic manifestations has been observed [4,5,11,12]. The most severe phenotypes occur in patients with frameshift or nonsense mutations in the N-terminal domain, including the Forkhead domain, while milder phenotypes associate with *FOXG1* missense mutations in the Forkhead domain [5]. As this genetic variability in patients makes it difficult to pinpoint the direct and indirect outcomes of identified mutations in the *FOXG1* gene, the challenge remains to dissect genotype–phenotype associations. The functional variability of residual FOXG1 protein and the contribution of dosage-effects to the syndrome make it even more difficult to disentangle the etiology of the syndrome and the diverse molecular functions of FOXG1. In this review, we discuss the clinical features and possible correlation to the seemingly diverse molecular alterations underlying FOXG1 syndrome, and explore up-to-date disease models, aiming to advance potential therapeutic avenues.

## 2. Clinical Manifestations of FOXG1 Syndrome

The first heterozygous de novo translocation mutation in *FOXG1* was identified in a 7-year-old patient in 2005 [13]. The patient manifested microcephaly, complete agenesis of the corpus callosum, and cognitive disability. Shortly after, two other individuals fulfilling the criteria for Rett syndrome variants were diagnosed carrying mutations in the *FOXG1* gene [14]. Due to a substantial phenotypic overlap between the condition caused by mutations in the *FOXG1* gene and RTT, mutations in *FOXG1* were classified as a congenital variant of Rett syndrome [14,15]. However, the increasing numbers of patients diagnosed with *FOXG1* mutations today, have allowed refinement of the phenotypic manifestations and classification of this condition as FOXG1 syndrome. Among the phenotypes of FOXG1 syndrome that diverge from typical Rett syndrome were the earlier (congenital) onset, specific brain imaging abnormalities, dyskinesia, and lack of regression, which comprise the hallmarks used to distinguish FOXG1 syndrome from RTT [4,5,11]. Subsequent studies and several clinical screens laid a further foundation to specify FOXG1 syndrome, which is now recognized as a distinct human neurodevelopmental disorder.

Despite the significant variability in phenotype and severity observed in individuals depending on the genotype, the core FOXG1 syndrome phenotype consists of severe postnatal microcephaly and mental retardation, deficient language development, poor social interactions and eye contact (indicating a link to ASD), postnatal growth deficiency, problematic sleep patterns, epilepsy, and irritability during infancy [4,12]. Additionally, data from brain imaging studies uncovered corpus callosum agenesis and reduced white matter, as well as poor and delayed myelination patterns [4,5,11].

**Figure 1:**
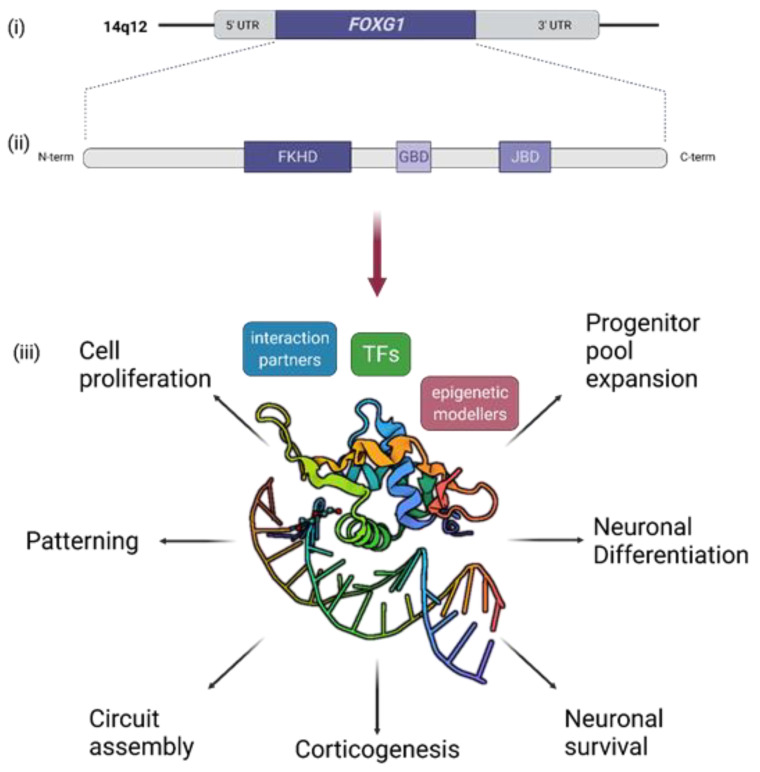
Functions of FOXG1. *FOXG1* is located in 14q12 in humans and contains only one exon (**i**). FOXG1 protein domains: FOXG1 consists of a Forkhead domain (FKHD), a 20-residue Groucho (Gro)-binding domain (GBD), and a 10-residue histone demethylase (KDM5B)-binding domain (JBD) (**ii**). FOXG1 plays important roles in many neurodevelopmental processes through its interaction with DNA and protein interaction partners (**iii**). C-term: C-terminus; N-term: N-terminus; TF: transcription factor; UTR: untranslated region. Illustration was created with Biorender.com.

In humans, *FOXG1* is located on chromosome 14q12 and contains only one coding exon [16,17]. Among vertebrates, the N-terminal domain does not display a large degree of evolutionary conservation [18]. In contrast, the amino acid sequence from the Forkhead domain (FKHD) to C-terminal domain is highly conserved [16,19]. The FKHD mediates binding of FOXG1 to the DNA. In addition to the FKHD, the FOXG1 protein harbors a 20-residue Groucho (Gro)-binding domain (GBD) and a 10-residue histone demethylase (KDM5B/JARID1B)-binding domain (JBD) within the C-terminal part (Figure 1). A striking feature of the N-terminal domain is its contribution to DNA-binding, in addition to the classical FKHD. Thereby, FOXG1 seems to recognize and bind to canonical FKHD recognition binding motifs and to alternative, non-canonical, DNA sequences [20,21,22]. Notably, other members of the FOX transcription factor (TF) family bind or recognize DNA through both their FKHD and the variable N-terminal protein domain, which indicates the important roles of other domains of the protein than the FKHD. However, these roles are not fully understood as of yet.

**Figure 2:**
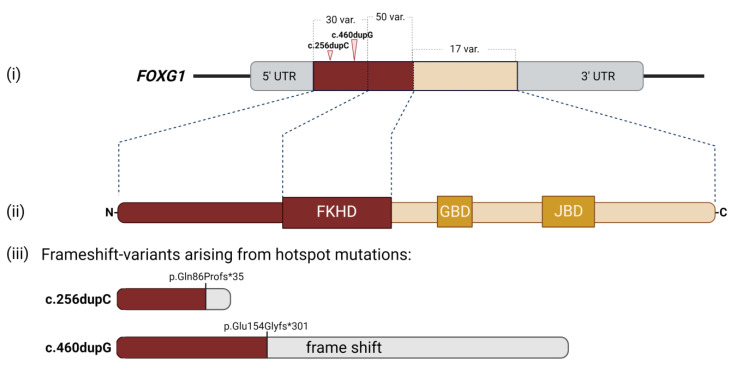
Mutation hotspots of FOXG1. *FOXG1* gene (**i**) and protein (**ii**) domains, and the distribution of variants in a schematic illustration depicting the N-terminal domain, Forkhead binding domain (FKHD), Groucho-binding domain (GBD), JARID1B-binding domain (JBD), and C-terminal domain. The mutations are distributed in all parts of the gene, affecting all protein domains. The most severe phenotypes are observed upon mutations in the N-terminal domain and FBD (shown in red) There are two mutation hotspots in the N-terminal region (arrows) that lead to frame shifts starting upstream of the FBD, GBD, and JBD. Potential variants arising from the mutations in the two hotspots, c.256dupC and c.460dupG, are shown in (**iii**). The variants in the C-terminal domain, including GBD and JBD, cause milder phenotypes of FOXG1 syndrome (shown in yellow). Number of variants observed in each protein domain are noted on the illustration [4,5,11,12]. UTR: untranslated region. Illustration was created with Biorender.com.

Since the first identified case of FOXG1 syndrome [13], around 860 patients carrying mutations in *FOXG1* have been diagnosed using chromosomal microarrays, whole exome sequencing (WES), and gene sequencing methods [1,4,5,7,11,12]. As mentioned before, *FOXG1* mutations encompass missense, nonsense, and frameshift mutations, as well as micro-deletions proximal to or spanning the *FOXG1* gene [4,5,8,9,11,15,23]. An updated listing of identified *FOXG1* mutations can be inferred from RettBASE (RettBASE. Available online: http://mecp2.chw.edu.au/foxg1/foxg1_variant_list_copy.php, accessed on 22 December 2021) [24,25]. The mutations are distributed throughout the entire gene and can, thus, localize to all known protein domains. However, a few hotspots were eminently more susceptible to de novo mutations, as has been reported by genotype–phenotype studies [1,4,5,11,12] (Figure 2). Two of these hotspots are located in stretches of the cytosine and guanine repeats within the 5′-end of the gene that encodes the N-terminal protein domain, c.256dupC and c.460dupG, respectively [4,5,11,26] (Figure 2). Mechanistically, one assumes a particular susceptibility towards replication errors in this genomic sequence [27]. In addition to mutations that occurred directly in the *FOXG1* gene, some patients carried microdeletions in genomic regions that regulate *FOXG1* expression. These deletions localized both up- and downstream of the coding sequence and mostly led to insufficient expression of *FOXG1* [8,9,28].

As screenings for FOXG1 syndrome became more prevalent, an increasing number of genomic variants and corresponding phenotypes have been identified, and this allowed the exploration of genotype–phenotype correlations in more detail. Several studies based on comparably large patient cohorts provided overlapping conclusions in regard to the phenotypic manifestation of FOXG1 syndrome. However, conflicting results regarding more uncommon phenotypes were also reported [4,5,11,29]. Most importantly, mutations in the 5′-end and the FKHD seemingly caused more severe phenotypes compared to mutations in other localizations. Notably, the most severe outcomes were observed in patients who carried mutations bearing truncated FOXG1 protein variants [5]. On the other hand, mutations located in the 3′-end of the gene and, thus, affecting the protein’s C-terminus were associated with milder phenotypes compared to the 5′-end. The high degree of evolutionary conservation of the FKHD among different species already hinted at its critical role in FOXG1 functions. It was thus expected that mutations affecting the DNA-binding domain of FOXG1 or the DNA-binding regulatory region within the N-terminal protein domain were poorly tolerated and led to severe phenotypes [5,21,22]. Puzzling, though, was the finding that the mutation hotspots of c.256dupC and c.460dupG associated with great variability of features and severities. This poor correlation between genotype and phenotype might, therefore, indicate that further components should be considered as potentially critical modulators of the clinical phenotypes in FOXG1 syndrome. Putative, as thus far mainly unexplored, components might include the genetic background, variable epigenetic landscapes, or environmental influences [5].

It has become clear that pleiotropic and non-redundant functions of FOXG1 are also involved in the severe pathophysiology, with complex genotype–phenotype relationships with mutations [4,5,12]. Thus, recognition of FOXG1 syndrome as a distinct entity was of great importance for focusing upcoming research efforts, disease modelling approaches, and subsequent potential therapeutic undertakings. Further activities should be based on a clear understanding of the diverse functions of FOXG1, especially in human model systems, to eventually embark on therapeutic avenues to cure or facilitate living with *FOXG1* mutations.

It seems that most therapies of FOXG1 syndrome have aimed at treating symptoms, including seizures. In this context, one study described the use of anti-epileptic drugs for RTT patients. A small minority of the patient cohort had a mutation in *FOXG1*, whereas the majority had typical Rett syndrome. The study did not differentiate between the typical RTT and FOXG1 syndrome, but reported the different responses of patients, which depended on the drug used and the age of the patients [30]. For the benefit of the patients, it is thus conceivable that new approaches should be explored to identify specified therapeutic targets and to reduce side effects. Approaches taken in this direction focused on small molecule screenings, RNA therapies, and antisense oligonucleotide (ASO’s) based medicine [31], but so far they have only resulted in a few publications, which will be discussed below.

## 3. Short Recapitulation of FOXG1 Functions in Brain Development and Function

FOXG1, previously also called brain factor-1 (BF-1), is a winged-helix TF of the Forkhead (FKH) family. FOXG1 is uniquely expressed in the nervous system, and is active in the early telencephalon, the cerebral cortex, and hippocampus, in addition to the inner ear, retina, and olfactory epithelium [2,18,32]. It has diverse and non-redundant functions, comprising cell proliferation and progenitor pool expansion [33], regional patterning of the forebrain [34], cell migration during corticogenesis [35] and circuit assembly [33,36,37,38,39] (Figure 1). Over the years, numerous studies, using conventional and conditional knockout mouse models and genome editing techniques, have established FOXG1 as a master regulator of embryonic and postnatal brain development, which has been recently reviewed in detail elsewhere [1,18]. Therefore, in this review, we focus on the functions and molecular aspects of FOXG1 relating to the core phenotypes of FOXG1 syndrome.

Transgenic mice are one of the best studied model systems to understand FOXG1 functions. Several *Foxg1* knockout mouse models were created by replacing the coding region of *Foxg1* with *lacZ*, *cre,* or *tet* (tetracycline transactivator) [32,33,40]. These mice all showed severe reduction in size of the cerebral cortex and mortality at birth [32]. Only the haploinsufficient *Foxg1^Cre/+^* mice survived postnatally, exhibiting microcephaly and impaired neurogenesis phenotypes in the cortex and hippocampus [41,42,43]. Therefore, the *Foxg1^Cre/+^* mice served as an appropriate model to study the human FOXG1 syndrome.

The mechanisms underlying the observed hypoplasia were investigated rather extensively. Cortical stem cells featuring constitutive loss of FOXG1 exhibited a premature lengthening of the cell cycle, concomitant with an increased exit from the cell cycle, which led to neuronal differentiation [32,33]. Both DNA-binding dependent and independent mechanisms regulated the functions of FOXG1 in cell cycle control. While the cell cycle length was dependent on a DNA-binding role of FOXG1, the normal cell cycle exit required that FOXG1 antagonize the anti-proliferative activity of TGFb by associating with DNA-binding proteins, which function as SMAD partners [33,44]. A decreased level of FOXG1 in intermediate progenitor cells (IPCs) was also associated with an increased expression of the cell cycle inhibitor *Cdkn1a* (p21), contributing to the early exit from the cell cycle [43]. Additionally, it was reported that FOXG1 antagonized FOXO/SMAD-dependent neuronal differentiation of cortical progenitors through direct association with the FOXO/SMAD complex, or by competitively binding the consensus FKH binding site [45,46]. FOXG1, thus, reduces the expression of *Cdkn1a*, prevents cell cycle exit, and enables the continued proliferation of FOXG1 expressing cells, consequently enabling prolonged progenitor pool expansion [33,45,46]. Cells lacking FOXG1 differentiate prematurely into neurons, thus depleting the progenitor pool and leading to a reduction in brain size. Together, the observed microcephaly in FOXG1-deficient mice was seemingly multi-faceted, and involved both DNA-dependent and independent mechanisms, which subsequently impinged on the regulation of cell cycle proteins and other factors.

As FOXG1 affected the early phase of corticogenesis, it was not surprising that layering defects were observed in *Foxg1* knockout mice. The mammalian cerebral cortex consists of six layers of neurons that are generated in an inside-out manner, except for the first-born neurons residing in layer 1, called Cajal Retzius cells (CRC) [47], reviewed in detail elsewhere [48,49]. In depth studies on the role of FOXG1 in corticogenesis and layering of the cortex used conditional knockouts of the murine *Foxg1*. Early on during corticogenesis, the absence of FOXG1 caused an excessive production of CRC, and a failure to produce later-born neurons [33,36]. Moreover, deep-layer progenitors reverted to the production of the early-born CRC [36]. Under normal conditions, FOXG1 coordinated the production of different types of projection neurons, through direct inhibition of the early progenitor transcriptional factor network of *Tbr1, Dmrta1, Ebf2,* and *Ebf3*, as shown by transcriptome and ChIP-seq studies [34,36,37]. Moreover, the expression of FOXG1 in the intermediate zone in the later stages of corticogenesis was required for the separation of later-born subtypes of cortical neurons [38]. Thus, FOXG1 has crucial roles in mammalian cortical expansion, not only in progenitor pool expansion but also in the proper layering and patterning of the cortex and subtype identities, consequently affecting the functionality of the cortex.

Agenesis of the corpus callosum is another hallmark of FOXG1 syndrome relating to cortical development, seen in varying severities in patients [4,13]. This malformation was also observed in some *Foxg1* haploinsufficient mice, while severe hypogenesis was a feature in *Foxg1* knockout mice [32,33,39,42]. Callosal projections connect both cerebral hemispheres and confer associative connections, disturbance of which was also observed, for example, in ASD. Neurons residing in all cortical layers contribute to callosal projections [50]. Although *Foxg1* expression becomes variable in post-mitotic neurons, recent studies have demonstrated that heterozygous deletion of *Foxg1* in mature cortical projection neurons resulted in defects in upper-layer projection neurons, concomitant to aberrant axonal projections through the corpus callosum [38,39]. Mechanistically, FOXG1 repressed NR2F1 (COUP-TFI) expression, which transformed local projection neurons to callosal projection neurons [38]. Additionally, FOXG1 formed a repressive complex with ZNF238 (RP58, ZBTB18), and ChIP-seq analyses revealed *Robo1*, *Slit3*, and *Reelin* as target genes of this repressor complex, all of which are key regulators of callosal axon guidance. Thus, FOXG1 plays a crucial role in establishing callosal projections and promotes the radial migration of cortical neurons [39]. These studies provided critical insight into the molecular mechanisms behind the agenesis of the corpus callosum, and identified FOXG1 as an important factor favoring cortico–cortico projections.

Over 80% of FOXG1 syndrome patients presenting with deletions or intragenic mutations of *FOXG1* are diagnosed with epilepsy, rendering this feature as another core phenotype [26]. Notably, patients with *FOXG1* duplications also developed epilepsy, albeit to a lesser extent [26]. Thus, deciphering the varying characteristics of epilepsy is important for distinguishing genotype–phenotype associations in patients, and to provide effective therapies. Epileptic phenotypes and seizures were also observed in *Foxg1^Cre/+^* mice. In vivo electrophysiological characterization of this animal model revealed that *Foxg1^Cre/+^* mice showed hippocampal hyperexcitability and that they were susceptible to seizures, which was linked to decreased expression of the chloride transporter KCC2 and the GABA transporter vGAT [51]. Similar insights were obtained for the *Foxg1* haploinsufficient cortex, whereby decreased levels of FOXG1 led to higher excitability and depressed synaptic transmission, due to increased expression of vGLUT2, accompanied by decreased levels of KCC2, and decreased levels of GLUA1 and PSD-95, respectively [52]. *Foxg1^Cre/+^* mice had susceptibility to seizures both in the cortex and the hippocampus, which were linked to excitation/inhibition imbalance. However, further underlying mechanisms remain to be unraveled.

Another method of action of FOXG1 impinged on mitochondrial function to regulate bioenergetics. This finding implicated that FOXG1 is crucial for mitochondrial functions during embryonic development and in pathological conditions [53], and further signified that it functions beyond chromatin-mediated transcriptional regulation. Interestingly, a triheptanoin-based anaplerotic diet, which has been used previously to treat some inherited metabolic disorders, including typical RTT [51,54,55,56], rescued the altered expression of KCC2 and vGAT, and normalized enhanced susceptibility to seizures [51]. Although we are still far from fully understanding the underlying mechanisms or the role FOXG1 plays in neuronal metabolism, and whether this translates to misbalanced excitation and inhibition, these findings present a promising therapeutic approach for alleviating the epileptic symptoms of FOXG1 syndrome patients.

Along these lines, in haploinsufficient *Foxg1* mice, GLUD1 (orphan glutamate receptor δ-1 subunit) expression decreased, alongside other GABAergic and glutamatergic markers, indicating a shift in excitation/inhibition balance [57]. Using cellular and animal models, this comprehensive study also reported a temporal shift towards a general decrease of brain synapses, although the regulatory link between FOXG1 and GLUD1 remains to be disentangled [57]. Additionally, FOXG1 associated with the microprocessor complex through its interaction with DDX5 and played a role in miRNA biogenesis of the miR-200 family regulating PRKAR2B expression post-transcriptionally. PRKAR2B inhibits postsynaptic functions by interfering with the PKA activity [58], implying that deregulation of PRKAR2B in FOXG1 syndrome could have contributed to the synaptic dysfunctions observed in patients.

Together, the diverse functions of FOXG1 at different developmental stages emphasize its importance in the proper development and function of the CNS, and establishes FOXG1 as a key player in human neurodevelopment, while the diverse mechanisms used to fulfill its functions remain to be fully explored.

In this context, it is important to mention that the critical role played by FOXG1 during early development of the brain brings other complications in applying various treatment options. Even if FOXG1 syndrome could be diagnosed prenatally, reverting phenotypic features in utero is very challenging. However, despite the fact that FOXG1 syndrome patients might not benefit immediately from insights coming from basic research, understanding the molecular etiology of FOXG1 syndrome might identify novel therapeutic targets and new resources of drugs aiming to alleviate some symptoms of the patients.

## 4. Human Cell-Derived Models of FOXG1 Syndrome and Function

While the constitutive knockout of *Foxg1* caused prenatal death in mice [32], its haploinsufficiency only exhibited mild microcephaly and behavioral abnormalities [36]. However, humans with a heterozygous loss of *FOXG1* develop variable symptoms, with differing severities [4,5,11,12]. Nevertheless, direct and deeper analyses of the effects of *FOXG1* mutations in humans is limited ethically to medical imaging, clinical observations, and post-mortem analyses. Therefore, developing appropriate models for projected investigations is crucial. Investigations during the last decades opened the door to additional opportunities for modelling, especially owing to the outstanding progress in stem cell biology. Human-induced pluripotent stem cells (hiPSCs) harbor a high potential for the study of neurodevelopmental diseases. Their use in basic research aims to decipher the molecular alterations underlying, for example, CNS diseases. This model system will provide an extended picture of potential mechanisms triggered by gene defects in human cells and allows for experimental attempts that bypass interspecies variations. Additionally, hiPSCs offer patient-specific modelling and therapeutic adjustments for many different diseases, as they are generated directly from patient-derived fibroblasts or other cells. They also mirror, more or less, the complete human neurodevelopmental process, starting from stem cells, passing through different progenitor steps, towards mature neurons and neuronal networks. This provides the opportunity to follow the temporal dynamics of FOXG1 expression during development in different cell types. During recent years, research on Rett syndrome, in the context of understanding the neurodevelopmental basis, has used hiPSCs and hiPSC-derived NSCs, as well as neurons, to analyze and describe changes in gene expression, cell activity, and cell composition [59]. The differentiation of RTT-patient-derived hiPSCs into neurons led to fewer synapses, reduced spine density, and smaller soma size through reduced MECP2 expression [60]. Moreover, comparative studies focusing on typical and atypical Rett syndrome have been fueled by hiPSC-derived technology. A comparison of molecular alterations between Rett syndrome caused by either *MECP2* or *CDKL5* mutation revealed common targets, including Glutamate Dehydrogenase 1 (GLUD1), which is encoded by the *GRID1* gene. Increased expression levels of GLUD1 were observed in NSCs differentiated from one patient cell line with *MECP2* mutation and two cell lines with *CDKL5* mutations [61].

In contrast to typical Rett syndrome, only a few studies have reported so far on FOXG1 syndrome patient-derived hiPSCs. In consequence, we are missing a rich data resource, to further discern molecularly and mechanistically between RTT, atypical Rett, and FOXG1 syndrome. Despite this general shortage of data reporting on molecular alterations in FOXG1 syndrome, the first studies are available, in which transcriptional alterations in hiPSC-derived (from two female *FOXG1*^+/−^ patients) NSCs were reported. The authors observed an imbalanced expression of the proteins that confer excitation and inhibition in patient-hiPSC-derived NSCs. This observation fostered the conclusion that FOXG1 is an important modulator of the ratio of excitatory and inhibitory neurons [57], similar to the outcomes from the mouse model described above [51]. Therefore, it seems highly likely that misbalanced neuronal activity in regard to excitation or inhibition is a direct link to the patient’s microcephalic and epileptic features. Interestingly, the same study also showed an increase of GLUD1 expression, similar to the observation of transcriptional alterations upon *MECP2* and *CDKL5* mutation, which hints towards commonalities between typical and atypical Rett and FOXG1 syndromes. Such overlaps might be particularly important for the rare diseases we are describing here, as they might be able to provide future therapeutic attempts [57]. However, GLUD1 was found to be decreased in mouse models. These contradicting findings between different model systems emphasize the power and essential utility of novel technologies such as hiPSCs to study FOXG1 syndrome in the best model of the patients’ conditions.

Thus, another promising avenue for using FOXG1 patient-derived hiPSCs and their cellular progeny is to test rescue strategies for the *FOXG1* mutation. Recently, the modification of patient cell lines using an adeno-associated virus (AAV)-coupled CRISPR/Cas9 system was reported [62]. This study showed that using CRISPR/Cas9-mediated genome editing was not only effective in repairing the mutation in primary fibroblasts of two FOXG1 syndrome patients, but also for correcting the pathogenic variant in hiPSCs. Effectivity on the molecular level was indicated, for example, by normalized levels of PAX6 expression in the developing neurons. With this study, the authors laid the foundation for a novel approach towards CRISPR-based personalized therapy of a severe neurodevelopmental disease [62].

Despite the low number of hiPSC-driven studies of FOXG1 syndrome, several hiPSCs that originate from FOXG1 syndrome patients are available for further research and are deposited in different biobanks [63], e.g., the Coriell Institute for Medical Research, USA or the Biobank of the University of Siena, Italy [64]. Current progress in stem cell research, especially regarding the generation of diverse types of brain organoids, gives additional opportunities to generate cellular model systems, in which, for example, dynamic spatiotemporal processes of early brain development can be mimicked. This renders stem cell research approaches very useful to model in particular human brain disorders [65]. Cerebral organoids can give important insights into neurodevelopmental diseases affecting the forebrain [66], and could, thus, serve as a suitable model and research platform to advance the understanding of FOXG1 syndrome, both mechanistically and clinically. As one of the first experiments in this direction, hiPSC lines and organoids from ASD patients led to the observation that increased levels of FOXG1 correlated with this disease [6]. Accordingly, treatment of these ASD mimicking organoids with different *FOXG1* shRNAs rescued the observed molecular changes. Levelling the FOXG1 expression towards control conditions, restored the differences in GABAergic neuronal differentiation that were observed in patients, compared to healthy donor-derived organoids. However, this rescue strategy did not majorly affect the expression of dorsal forebrain marker genes or transcription factors responsible for cortical excitatory neuron differentiation [6]. Together, this ground-breaking study revealed the important role of FOXG1 in controlling the balance of neuronal subtypes in functional neuronal networks [6].

Another line of experiments was based on CRISPR/Cas9 and small molecule-assisted shut-off (SMASh) technology to modulate *FOXG1* expression in hiPSCs and hiPSC-derived cells. This experimental paradigm allowed the investigation of FOXG1 function in a dosage-dependent setting, in which the differentiation of hiPSCs into neurons was studied, as well as the cross talk of cortical and medial ganglionic eminence organoids [67]. In accordance with the findings upon increased expression, the reduced expression of FOXG1 bore fewer inhibitory GABAergic neurons, alongside impaired maturation of this interneuron class and an overall smaller brain organoid formation. The severity of the phenotypic alterations upon impaired FOXG1 expression correlated directly with the remaining dose of FOXG1 in the cells, emphasizing that the well-balanced availability of the FOXG1 protein is critical for proper brain development and function [67].

While reports on hiPSC models of FOXG1 syndrome are still few in number, more studies focused on typical Rett syndrome patient-derived hiPSCs. As these were reviewed recently [59], and due to the primary focus of this review on FOXG1 syndrome, we only refer to some selected highlights, illustrating the advances in translational research using hiPSCs. Worth mentioning is that Rett syndrome was not only studied in 2D culture systems, but also in 3D organoids. RTT-mimicking brain organoids showed generally premature development of the cortical subplate, alongside mutation-specific changes [68]. Furthermore, such organoids had a highly abnormal and epileptiform-like activity, which was rescued by pifithrin-alpha, a neuroregulatory drug [69]. Of note, the suitability of brain organoids for screening for drugs to alleviate, for example, synaptic dysfunctions was illustrated in a recent report. This study identified two compounds, Nefiracetam and Carbamoylcholine, that rescued impaired synaptogenesis in MECP2-deficient organoids [70]. Interestingly, balancing overactive BMP signaling in RTT organoids using pharmacological inhibitors or specific miRNAs, alleviated differentiation defects [71,72]. As small RNAs are considered as biopharmaceuticals, for example to treat heart dysfunctions or cancers [73], their effectivity in treating RTT organoids might render them suitable to also treat features in other brain pathologies, including FOXG1 syndrome. However, comparable experimental approaches have not been undertaken as of yet for hiPSCs and organoid-derivatives with *FOXG1* mutations. Nevertheless, the insights gained already by the few RTT studies using brain organoids pave the way towards translational research for FOXG1 syndrome as well. The novel insights also emphasize the emerging importance of the organoid model system per se, to understand, for instance, the unresolved relation between individual genotypes and phenotypes in FOXG1 syndrome. In addition, brain organoids could serve to exploit patient-specific therapeutic options in diseases such as FOXG1 syndrome that have these highly variable features. Overall, there are now opportunities for investigating FOXG1 syndrome in hiPSCs and different types of organoids, e.g., modelling the ventral or the dorsal telencephalon and to verify and extend findings from mouse or other animal models. However, more importantly, these novel technologies allow obtaining more insights into the human- and mutation-specific mechanisms driving the FOXG1 syndrome phenotypes.

## 5. Future of FOXG1 Syndrome Modelling, Therapeutic Prospects, and Limitations

In all, patient-derived hiPSCs, as well as brain organoids generated from such resources, are seemingly very promising approaches for modelling neurodevelopmental diseases, including FOXG1 syndrome. While hiPSC 2D cultivation and differentiation serve as a model for the development of single cell lineages and to decipher cell lineage-dependent molecular mechanisms, 3D brain organoids add to this, as they represent a more systematic model, by integrating the single cells into a fairly natural network, which consists of a heterogeneous variety of cell lineages [74].

**Figure 3:**
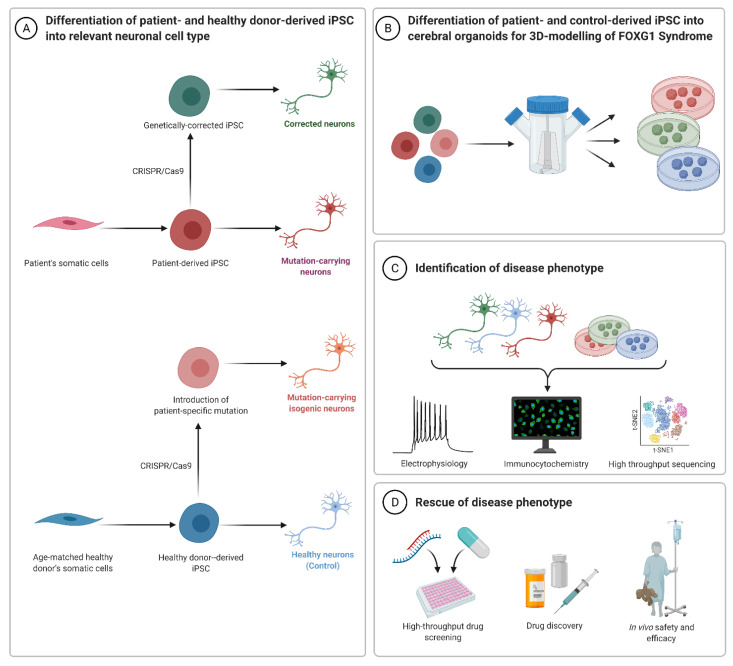
Approaches and potentials of human-based disease modelling for FOXG1 syndrome. (**A**) Upon genetic screening for FOXG1 syndrome, specific mutations are identified. Patient-derived somatic cells (fibroblast and other cell types) are reprogrammed to a pluripotent state (iPSC). The mutations in these cells are ‘corrected’ using a CRISPR/Cas9 approach. In parallel, iPSCs from healthy donors are used to introduce the patient-specific mutations using CRISPR/Cas9 genome editing approaches. iPSCs carrying the disease-related mutation can be differentiated into neural cell types that are affected in the disease. (**B**) In another approach, iPSCs carrying patient-specific mutations are used to generate cerebral organoids to model neurodevelopment. (**C**) High throughput sequencing approaches and functional assays are employed on these differentiated neurons and cerebral organoids to recapitulate the syndrome in the human model system. (**D**) Potential targets and biomarkers obtained from these studies would contribute to drug discoveries and treatment of symptoms in patients. Illustration was created with Biorender.com.

The hiPSCs technology will allow studying individual mutations of FOXG1 syndrome patients, in order to determine genotype–phenotype specific correlations, cellular processes, and molecular mechanisms. Additionally, the advantage of CRISPR/Cas9 editing is the ability to introduce specific mutations into an even more controlled model system, due to isogenic backgrounds. Therefore, studies on genome-edited hiPSCs might avoid the batch effects caused by the patient specific genomic or epigenetic background. Moreover, approaches resembling those of the AAV-mediated repair of *FOXG1* mutations not only serve the generation of cell lines with the same genetic background, but also lay a foundation for individualized medicine and the early gene-therapy of FOXG1 syndrome [62] (Figure 3).

Albeit promising, disease-modelling systems also have their limitations. Mice and other animals have the advantages that an entire organism and entire developmental processes can be studied, but they lack the human background, have limitations in mimicking all facets of human CNS development, and cannot reflect human-specific gene regulation and molecular mechanisms. Aspects such as the basic understanding of brain development in FOXG1 syndrome may be sufficiently mirrored, but systematic outcomes and whole-organism effects can only be conjectured. The same limitations, however, also apply to hiPSC-derived organoids. Nevertheless, the ongoing and future development of highly structured organoids, combining various CNS regions and containing blood-vessel structures, will give rise to even better model systems. Such advanced or second-generation organoids are closer to the natural development compared to the possibilities that are the current state-of-the-art. One can hope that in the future, organoids can grow further to mimic late human development and function, to include a higher variety of neural subclasses, and thus a more complex brain/organoid structure. The first generation of vascularized organoids [75,76] gives rise to further development, standardization, and generation of models reflecting later neurodevelopmental steps. The generation of vascularized organoids with a potentially functional blood–brain barrier [77], as well as the screening and analysis of potential drugs with blood–brain-barrier organoids [78], are promising and necessary steps towards therapeutic approaches. Moreover, the fusion of brain organoids is currently used to model interactions between different brain regions [79]. The development of additional tissues, such as bilateral optical vesicles [80], or even the fusion with muscle cells for the development of motoneurons and of neuromuscular junctions [81,82] have been performed, and these studies exemplified the advantages ahead in using organoids to study human brain diseases. Thus, these approaches will prove useful in the future to investigate in further detail how FOXG1 influences later neural development, its potential influence on tissue interaction, on entire organ function, and even multi-organ interplay. The use of such ‘higher developmental organoids’ will serve to decipher the fundamental functions of FOXG1 in neural development. In addition, they can be exploited as standardized model systems for screening potential drugs targeting symptomatic and developmental features of FOXG1 syndrome patients. Examples of similar approaches have been reported for Glioblastoma patients [83], amongst others [84,85]. In this light, personalized medicine for individual FOXG1 patients could be implemented in the future on the grounds of screening patient-derived organoids with common or new drugs.

Nevertheless, researchers should also be aware of the ethical issues that are associated with the generation of organoids with increasing complexity and developmental structures [86].

## 6. Conclusions

FOXG1 syndrome is a severe neurodevelopmental disorder that has been studied for many years as a variant of Rett syndrome, and must, therefore, be considered to be under-diagnosed as an individual disease. The diversity of *FOXG1* mutations, the plethora of FOXG1-associated mechanisms of transcriptional and posttranscriptional regulations, and the diverse and non-redundant functions of FOXG1 underline the importance of further research into disease-relevant alterations. Research focusing on the exploration of varying FOXG1-dependent molecular mechanisms on the one hand, and understanding genotype–phenotype correlations on the other hand, will lead to further discrimination of FOXG1 and other Rett-like syndromes. To date, many different studies have pointed out the importance of FOXG1 in spatiotemporal control of neurodevelopment, the development and composition of different neural lineages within the CNS, and the resulting effects on neural plasticity. These results can be directly connected to the severe phenotypes that are observed in FOXG1 syndrome patients. The current development in stem cell biology and disease modelling offers a variety of opportunities to investigate FOXG1 function and the individual genotype–phenotype relations even further. With the help of patient-derived hiPSCs and organoids, the development of new and personalized therapeutic approaches for FOXG1 syndrome can be improved and expedited. After an upcoming experimental phase, aiming to corroborate findings of animal models in human organoids harboring various complexities, this novel technology shall start to integrate the most important findings, and finally to pinpoint key players that one can exploit therapeutically to improve conditions for living with *FOXG1* mutation.

## Data Availability

Not applicable.
